# Design and content validation of the Transcend instrument for family caregivers

**DOI:** 10.15649/cuidarte.4595

**Published:** 2025-06-04

**Authors:** Kevin Julian Aya-Roa, Vicente Beltrán-Campos, José Angel Hernández Mariano, María de Lourdes García-Campos, Xóchilt Sofia Ramírez-Gómez, Carlos Alberto Núñez-Colín

**Affiliations:** 1 Doctoral student in Nursing Sciences, Universidad de Guanajuato, Health Sciences and Engineering Division, Celaya-Salvatierra Campus, Celaya, Mexico. E-mail: kj.ayaroa@ugto.mx Health Sciences and Engineering Division, Celaya-Salvatierra Campus Celaya Mexico kj.ayaroa@ugto.mx; 2 Full-time Professor, Universidad de Guanajuato, Clinic Nursing Department, Health Sciences and Engineering Division, Celaya-Salvatierra Campus, Celaya, Mexico. E-mail: vbeltran@ugto.mx Health Sciences and Engineering Division, Celaya-Salvatierra Campus Celaya Mexico vbeltran@ugto.mx; 3 Research Division, Hospital Juarez de Mexico, Mexico City, Mexico. E-mail: j_a_hm@hotmail.com Hospital Juarez de Mexico Mexico City Mexico j_a_hm@hotmail.com; 4 Full-time Professor, Universidad de Guanajuato, Clinic Nursing Department, Health Sciences and Engineering Division, Celaya-Salvatierra Campus, Celaya, Mexico. E-mail: lulu.garcia@ugto.mx Health Sciences and Engineering Division, Celaya-Salvatierra Campus Celaya Mexico lulu.garcia@ugto.mx; 5 Full-time Professor, Universidad de Guanajuato, Clinic Nursing Department, Health Sciences and Engineering Division, Celaya-Salvatierra Campus, Celaya, Mexico. E-mail: xs.ramirezgomez@ugto.mx Health Sciences and Engineering Division, Celaya-Salvatierra Campus Celaya Mexico xs.ramirezgomez@ugto.mx; 6 Full-time full professor, Health Sciences and Engineering Division, Celaya Salvatierra Campus, Universidad de Guanajuato, Mutualismo Head Office, Celaya, Mexico. E-mail: carlos.nunez@ugto.mx Universidad de Guanajuato Celaya Mexico carlos.nunez@ugto.mx

**Keywords:** Caregivers, Chronic Disease, Validation Study, Psychometrics, Nursing Theory, Cuidadores, Enfermedad Crónica, Estudio de Validación, Psicometría, Teoría de Enfermería, Cuidadores, Doença Crônica, Estudo de Validação, Psicometria, Teoria de Enfermagem

## Abstract

**Introduction:**

Transcendence is a metaphysical phenomenon of the self that is reflected in caregivers by transforming their attitudes, perceptions, and caregiving meanings about the care recipient and themselves.

**Objective:**

To develop and validate the content of the "Transcend" instrument through expert judgment and to determine its preliminary reliability.

**Materials and Methods:**

This methodological study focused on the design and content validation, via expert judgment, of an instrument to measure family caregiver transcendence called "Transcend," following the methodology proposed by Waltz. The judges assessed instrument's clarity, coherence, and relevance. After validation, the instrument was administered to 30 caregivers to assess its clarity in the population and its preliminary reliability.

**Results:**

Fifteen judges participated in the validation process, and a significant level of agreement was found (p < 0.001) in assessing clarity, coherence, and relevance. The content validity index, both individual and global, was 1.00. All items obtained an Aiken's V value ≥ 0.69, with values falling within the confidence intervals. Preliminary reliability in the pilot testing yielded a Cronbach's alpha of 0.90.

**Discussion:**

Validity and concordance indices should be interpreted together to determine the level of content validity based on expert judgment.

**Conclusion:**

The Transcend instrument shows adequate content validity and high preliminary reliability, supporting its progression to a second phase of validation, which will be crucial to determining its potential as a tool for assessing transcendence in family caregivers.

## Resumen

**Introducción:**

La trascendencia es un fenómeno metafísico del ser que se refleja en los cuidadores al transformar sus actitudes, percepciones y significados del cuidado, del sujeto cuidado y de sí mismos.

**Objetivo:**

Diseñar y validar, mediante juicio de expertos, el contenido del instrumento "Trasciende" y conocer su confiabilidad preliminar.

**Materiales y métodos:**

Estudio metodológico centrado en el diseño y la validación de contenido, mediante juicio de expertos, de un instrumento de medición de la trascendencia del cuidador familiar llamado “Trasciende”, siguiendo la metodología propuesta por Waltz. Los jueces evaluaron la claridad, coherencia y relevancia del instrumento. Tras la validación, se aplicó a 30 cuidadores para evaluar la claridad en la población y su fiabilidad preliminar.

**Resultados:**

Quince jueces participaron en la validación, encontrándose una concordancia significativa (p < 0.001) en la evaluación de claridad, coherencia y relevancia. El índice de validez de contenido, tanto individual como global, fue de 1. Todos los ítems obtuvieron un valor de V de Aiken ≥ 0.69, y dentro del intervalo de confianza. La confiabilidad preliminar en la prueba piloto arrojó un alfa de Cronbach de 0.90.

**Discusión:**

Los índices de validez y concordancia deben interpretarse juntos para determinar el nivel de validez de contenido basado en el juicio de expertos.

**Conclusiones:**

El instrumento Trasciende evidencia una adecuada validez de contenido y alta confiabilidad preliminar, permitiendo avanzar en una segunda fase, la cual será crucial para determinar el potencial como herramienta para valorar la trascendencia en cuidadores familiares.

## Resumo

**Introdução:**

A transcendência é um fenômeno metafísico do ser que se reflete nos cuidadores ao transformar suas atitudes, percepções e significados do cuidado, do sujeito cuidado e de si mesmos.

**Objetivo:**

Desenhar e validar, por meio de julgamento especializado, o conteúdo do instrumento "Trasciende" e determinar sua confiabilidade preliminar.

**Materiais e métodos:**

Estudo metodológico centrado no desenho e validação de conteúdo, por meio de julgamento de especialistas, de um instrumento de medida da transcendência do cuidador familiar denominado "Trasciende", seguindo a metodologia proposta por Waltz. Os juízes avaliaram a clareza, coerência e relevância do instrumento. Após a validação, foi administrado a 30 cuidadores para avaliar sua clareza na população e sua confiabilidade preliminar.

**Resultados:**

Quinze juízes participaram da validação, encontrando concordância significativa (p < 0,001) na avaliação de clareza, coerência e relevância. O índice de validade de conteúdo, tanto individual quanto global, foi 1. Todos os itens obtiveram valor de V de Aiken ≥ 0,69, e dentro do intervalo de confiança. A confiabilidade preliminar no teste piloto produziu um alfa de Cronbach de 0,90.

**Discussão:**

Os índices de validade e concordância devem ser interpretados em conjunto para determinar o nível de validade de conteúdo com base no julgamento de especialistas.

**Conclusões:**

O instrumento Trasciende apresenta adequada validade de conteúdo e alta confiabilidade preliminar, permitindo avançar para uma segunda fase, que será crucial para determinar o potencial como uma ferramenta para avaliar a transcendência em cuidadores familiares.

## Introduction

Non-communicable diseases, also known as chronic non-communicable diseases, are a group of long-term illnesses that are not caused by acute infections and can significantly affect long-term health. These diseases often require continuous treatment and ongoing care for effective control^1^,^2^. 

According to the World Health Organization (WHO), the burden of chronic diseases, such as cardiovascular, respiratory, endocrine, neurological, and kidney conditions, increased in the Americas between 2000 and 2019. These diseases have become leading causes of morbidity, mortality, and disability in the American region^1^-^6^. 

The rising burden of chronic diseases, combined with the shifting demographics of the Hispanic population, may lead to increased demand for home care, which in turn could intensify unpaid health care work (TNRS in Spanish)^7^-^10^. 

In Mexico, TNRS represents the caregiving activities performed by family members to prevent diseases and maintain the health of other relatives, whether at home or in other households. Estimates show that in 2021 alone, 55.6% of all TNRS in the country was dedicated to caring for individuals with chronic conditions and disabilities, which is equivalent to approximately 33,127 hours of care^7^,^11^. TNRS affects not only caregivers' autonomy but also their quality of life^10^. 

The demand for family caregivers is expected to increase significantly as new generations age. As a result, the number of adults who will require care is estimated to increase by 75%^12^. 

Family caregivers are defined as individuals who provide informal care, devoting time and effort to caring for a close relative without receiving any remuneration. In most cases, this role is assumed by someone with a familial or emotional bond, such as a spouse, child, or other close relative^13^,^14^. 

Family caregivers do not always assume their role consciously when a person with a chronic illness reaches a level of dependency. In many cases, caregiving begins gradually and unwittingly from the time of diagnosis, as a close family member starts to assume responsibilities such as selecting or preparing healthy meals, encouraging physical activity, accompanying the person to medical appointments, reminding them to take medication, and performing various supportive tasks^15^. 

Each caregiving experience is unique and varies depending on multiple factors, including the type of chronic illness, the level of dependency, and the age of the person receiving care. However, the caregiving process begins when a family member, either consciously or unconsciously, assumes or is assigned this responsibility. 

Caring for a chronically ill person at some point becomes an experience that often exceeds the caregiver's physical and mental capacities, restricting not only activities of daily living but also disrupting routines and affecting religious and spiritual practices^16^,^17^. 

When caregiving responsibilities interfere with activities related to personal and spiritual connection, they hinder the possibility of transcending, that is, the possibility of improving the caregiving relationship with oneself, one’s belief system, and the person receiving care. This disruption can negatively affect family relationships, weakening the bond between caregiver and cared-for person and contributing to the onset of depressive symptoms, affecting the caregiver's well-being and quality of life^18^-^21^. On the other hand, although many caregivers experience a great burden due to their functions, they also identify positive aspects of their role. Caregiving offers them opportunities to discover or deepen a sense of life purpose, foster a sense of personal satisfaction, enhance adaptability to different situations, and strengthen interpersonal relationships^22^, a phenomenon we refer to as Family Caregiver Transcendence. 

**Family Caregiver Transcendence**

The concept of "family caregiver transcendence," which underpins the measurement instrument presented here, is derived from the theoretical construct of self-transcendence proposed by Pamela Reed in her theory. This construct is defined as the expansion of perceptions, concepts, and meanings in a multidimensional manner, either through personal experience or connection with others. It is also understood as a dynamic process in which one's perception extends beyond immediate, limited perspectives held at a given moment^23^. 

Transcendence, as a phenomenon, belongs to the ontological and metaphysical dimensions of being, where the mind is integrated with each person's spirituality. It is expressed through the search for meaning or significance, particularly in risk situations. This is how transcendence can become a human capacity to identify one's own vulnerability, reflect on it, and seek a solution by finding meaning in one's own existence^24^. 

Transcendence can be defined as an unfolding process that entails a shift in perspective—from a predominantly rational and materialistic worldview to a broader conception of the world. This shift is marked by the expansion of personal boundaries across four key dimensions: intrapersonal, interpersonal, transpersonal, and temporal. Intrapersonally, transcendence is expressed through greater self-awareness, self-acceptance, a sense of coherence, and ego integration. Interpersonally, it is reflected in the ability to form deeper connections with others and nature. Temporally, it involves integrating the past and future into a meaningful and fulfilling present. Transpersonally, it is manifested in a connection with spiritual dimensions and aspects of the world that go beyond the perceptible. This process not only deepens one's understanding of the self but also enriches the overall life experience by integrating multiple dimensions of existence^23^-^26^.

To transcend is to serve a purpose greater than oneself, guided by a selfless intention and a desire to serve^27^,^28^. For men, caregiving can become a source of personal growth and fulfillment, providing a more satisfying experience and supporting continuity. For women, on the other hand, spirituality often plays a fundamental role in coping. Both men and women develop skills that enable them to achieve higher levels of adaptation^29^. Thus, the caregiving experience not only promotes intrapersonal and transpersonal (spiritual) growth but also becomes an opportunity for transcendence. As care needs evolve, the relationship quality becomes crucial to the experience^29^. Transcending within a special bond involves generating transformative meaning and a special relationship with the cared-for family member. This connection transcends the physical, expanding mutual understanding, empathy, and shared purpose. During this process, the caregiver's role is redefined, encouraging a lasting commitment to the well-being of the care recipient and strengthening the caregiver's capacity to navigate the challenges inherent in the role^30^. 

It is important to note that transcendence is an attribute of spirituality. Caregivers who strengthen their spirituality are able to overcome challenges and find transformational meaning in their caregiving activities, a phenomenon referred to as family caregiver transcendence. Although spirituality is often mistaken for religiosity, religious practices, or divine and magical beliefs, it encompasses personal and interpersonal aspects, allowing every person who strengthens his or her spirituality to achieve transcendence, which is reflected in improved interpersonal relationships and an expanded view of the world and life^31^,^32^. 

**Measuring instruments**

In previous review studies on instruments used to assess metaphysical phenomena, no specific tool was found for assessing transcendence in caregivers. There are three scales that measure this concept from a theocentric perspective, focusing exclusively on connection to a belief system, God, or a higher power, while overlooking other attributes of the phenomenon^33^-^38^. 

The Assessment of Spirituality and Religious Sentiments (ASPIRES) Scale consists of 39 items divided into two subscales. It was designed to assess spirituality as a sixth dimension of personality, in alignment with the Five-Factor Model (FFM) in psychology. The spiritual transcendence subscale includes 23 items and measures spiritual transcendence as a person's effort to give a deeper meaning to his or her life. This subscale comprises three dimensions: (I) Prayer Fulfillment, defined as the ability to create personal space for connecting with a higher reality; (II) Connectedness, understood as a sense of belonging to a transcendent reality that spans across different groups and generations; and III) Universality, which reflects the belief in a greater purpose in life that transcends everyday knowledge^39^. 

The Adult Self-Transcendence Inventory (ASTI) measures transcendence as a developmental process leading to wisdom and adaptation in adulthood. This process is characterized by an increasing ability to detach from social roles, achievements, and material possessions, thereby promoting greater integration and the dissolution of boundaries between the self and others. This instrument includes two dimensions —self-transcendence and alienation— and demonstrated satisfactory internal consistency (ω = 0.96 and ω = 0.93)^40^. 

Pamela Reed's Self-Transcendence Scale consists of 15 items rated on a Likert scale to assess self-transcendence. Its reliability, measured by Cronbach's alpha coefficient, ranges from 0.8 to 0.94 in the original versions. Regarding the translation into Spanish, fit analyses indicate the presence of a single factor^41^,^42^. However, a discrepancy exists between the multidimensional theoretical concept of self-transcendence and its empirical representation, suggesting a disconnect between the construct and its practical measurement. 

Therefore, designing and validating an instrument to assess transcendence in family caregivers grounded in nursing theoretical frameworks is necessary. Such a tool not only bridges theory and practice but also serves as a tool to guide future research. This study is part of a larger research project aimed at developing the conceptual and instrumental basis of family caregiver transcendence. In this phase, the objective was to design and validate the content of the measurement instrument, titled "Transcend," through expert judgment. Additionally, the study sought to know the instrument's preliminary reliability in the target population. 

## Materials and Methods

**Study design**

**Figure 1:**
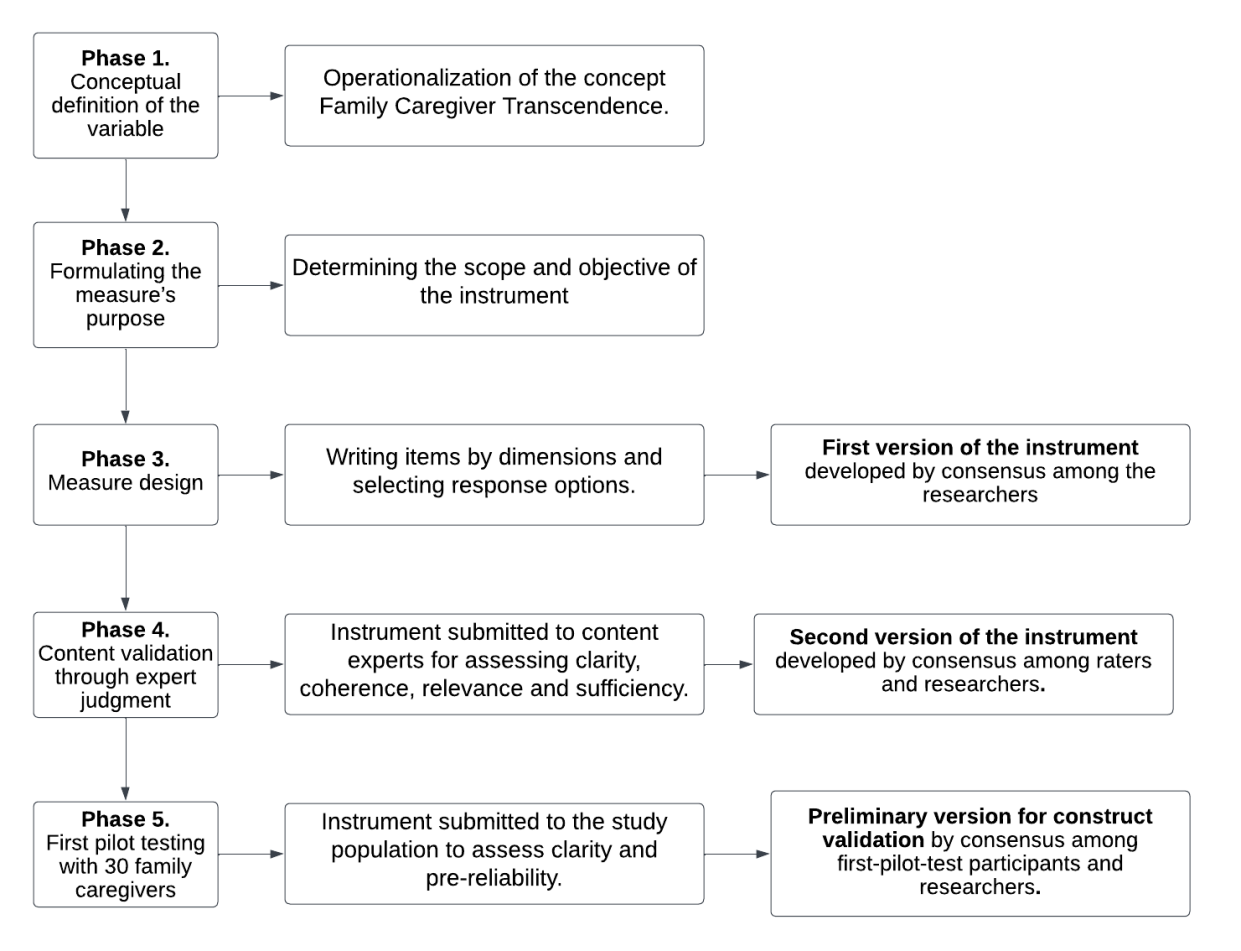
Methodological design

 This methodological study focused on the design and content validation, through expert judgment, of an instrument to measure family caregiver transcendence, titled "Transcend," and the methodology proposed by Waltz^43^. Five phases were followed in this study detailed in Figure 1.

**Figure 2:**
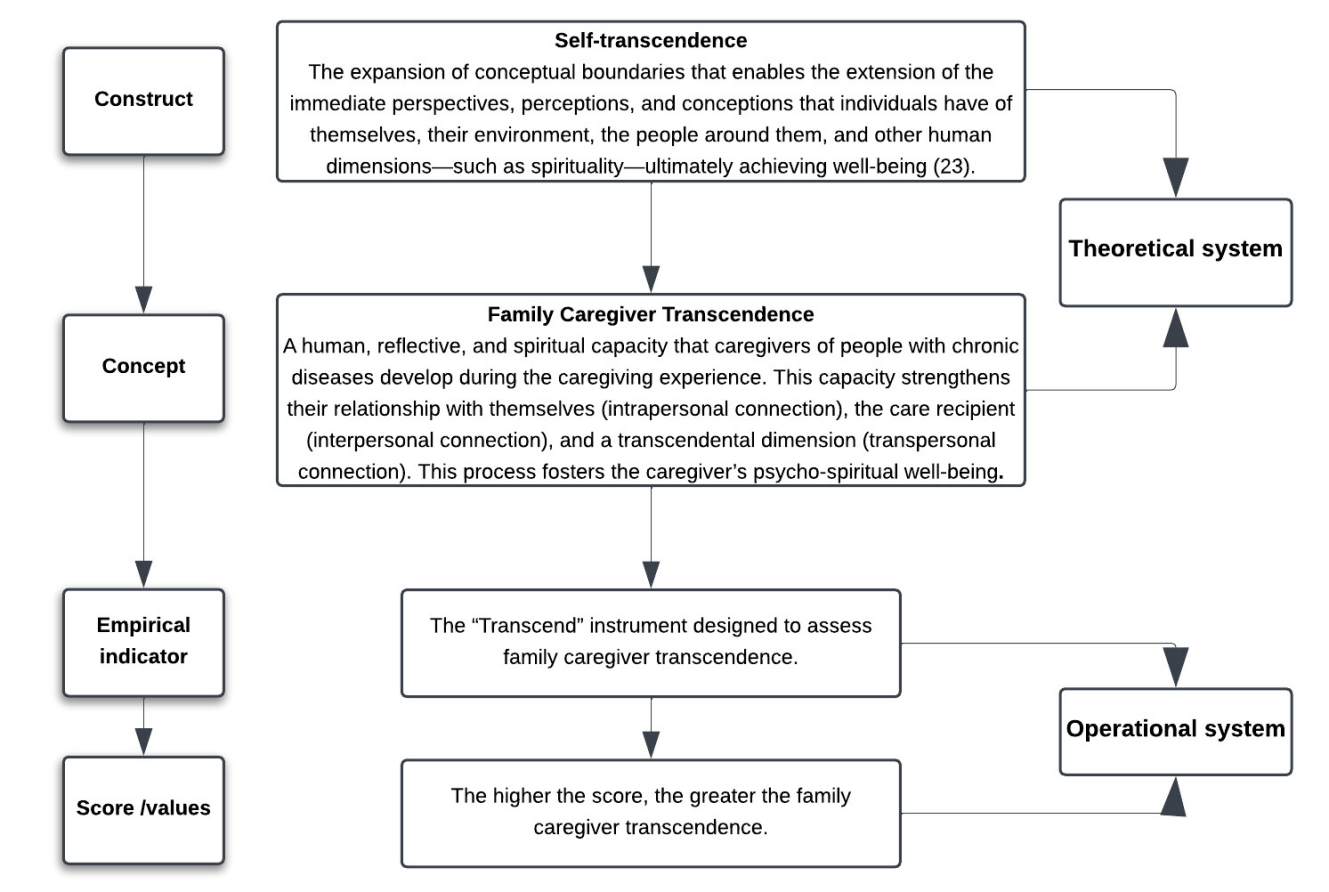
Concept operationalization of family caregiver transcendence

**Phase 1. Conceptual definition of the variable**

It is essential to have a clear and precise definition of the concept to measure a phenomenon. This involves concept operationalization, which is the process of delineating how a concept will be measured in terms of the observable indicators associated with it through dimensions or attributes^43^. For this process, the substruction strategy proposed by Dulock and Holzemer^44^ was applied to define the concept based on the concept of self-transcendence from Pamela Reed's theory^23^ (See Figure 2). 

**Phase 2. Formulating the measure's purpose**

In this phase, the objective of the instrument was established, which was to assess family caregiver transcendence by measuring caregivers' relationship with themselves (Dimension I: Intrapersonal connection), with the care recipient (Dimension II: Interpersonal connection), and with nature or a supreme being (Dimension III: Transpersonal connection). This instrument will provide insight into the capacity of family caregivers of individuals with chronic illness to experience transcendence. 

**Phase 3. Measure design**

Before designing the measure, a scoping review^38^ was conducted to identify different definitions and operationalizations of transcendence and similar constructs. The psychometric properties and specific characteristics of the instruments reviewed were also analyzed. Among the findings, it was observed that most items were formulated as affirmative statements, typically ranging from 15 to 23 items per instrument. Additionally, the most commonly used evaluation method was the level of agreement, assessed through Likert-type scales. 

Based on the operationalization of the concept and the literature review, transcendence is understood as a human capacity, both reflective and spiritual, that emerges from the experience of caring for a family member with chronic disease. Transcendence manifests across three dimensions: intrapersonal —the caregiver's relationship with themselves; interpersonal —the relationship with the care recipient; and transpersonal —the connection with a supreme being or nature. Each dimension encompasses distinct characteristics. Based on this conceptualization, ten affirmative items were formulated for each dimension, and, based on their characteristics, a five-point Likert-type scale was selected as the response format (strongly disagree, disagree, neither agree nor disagree, agree, strongly agree). Items 1 to 10 correspond to the intrapersonal connection dimension, items 11 to 20 to the interpersonal connection, and items 21 to 30 to the transpersonal connection. The resulting instrument has been named "Transcend" (see Table 1). 

**Phase 4. Content validation through expert judgment**

A search for scientific literature was conducted in the LILACS and Virtual Health Library (VHL) databases using the keywords self-transcendence, transcendence, nursing, caregivers, chronic disease, and psychometrics. Several researchers were identified. Their curricula vitae and email addresses were searched, and 15 expert judges were selected who met the following criteria:

- Nurses with postgraduate education (master's or doctoral degree).

- Nurses with knowledge of Pamela Reed's theory of self-transcendence and at least one scientific publication on the topic.

- Experienced in primary care nursing research.

- Experienced in the validation of measurement instruments.

- Fluency in Spanish.

**Table 1:** Dimensions of family caregiver transcendence, definitions, and items

Dimensions and definitions	Items
**Intrapersonal connection:** The relationship the caregiver develops and strengthens with themselves throughout the caregiving experience. This connection is characterized by a sense of personal growth, increased self-knowledge, and a deep state of reflection that enriches life purpose, self-care, and recognition of their needs. It also encourages continuous learning to manage stress and face the daily challenges of being a caregiver.	1. My experience as a caregiver has allowed me to get to know myself better.
	2. Providing care to a chronically ill person has led me to learn more about my emotions and feelings.
	3. My experience as a caregiver has helped me find purpose in my life.
	4. Caring for a chronically ill person has led me to reflect on my life and take care of my health.
	5. In my experience as a caregiver, I have learned to value my life more.
	6. Caring for a chronically ill person has led me to learn more about my own needs.
	7. I have learned to manage stressful, distressing, or emotionally charged situations associated with caregiving.
	8. I allow myself to take breaks or time off without feeling guilty.
	9. I regularly make time for activities that benefit me personally.
	10. I have learned to deal constructively with the challenges I may face as a caregiver.
**Interpersonal connection:** The relationship the caregiver develops and strengthens with the family member with chronic disease during the caregiving experience. This relationship is characterized by clear and effective communication, mutual respect, and a constant effort to provide the best possible care. It also includes offering emotional and spiritual support, as well as the ability to maintain a stable relationship despite the demands and challenges of caregiving. Through this process, empathy deepens, and the caregiver–care recipient bond becomes a special and meaningful connection.	11. Thanks to the caregiving experience, I have been able to improve my relationship with my family member with chronic disease.
	12. I enjoy sharing with my family member with chronic disease.
	13. Communication with my family member is clear and good all the time.
	14. I treat my family member with respect at all times.
	15. Despite the illness, I make plans for the future with my family member.
	16. I try to take care of my family member to the best of my ability.
	17. The emotional and spiritual support I provide to my family member is constant.
	18. I understand the needs that my family member with chronic disease may have.
	19. Despite the difficult and vulnerable moments I may experience as a caregiver, I strive to maintain a personal, stable, and harmonious relationship with my family member.
	20. I feel emotionally close and connected to my family member with chronic disease, which motivates me to provide better care.
**Transpersonal connection:** The relationship the caregiver develops and strengthens with a supreme being, the divine, or nature during the caregiving experience. This connection is characterized by strengthening their belief system and spirituality, where faith becomes a coping mechanism in facing caregiving challenges. Through religious, spiritual, or nature connection practices, caregivers find comfort, support, and motivation to continue their work. In addition, this relationship fosters deep reflection on the meaning of life, death, and existential purpose, which not only enriches their caregiving experience but also enables them to provide more compassionate and meaningful care.	21. Caring for another person has strengthened my connection to God, a higher power, or nature.
	22. My spirituality has given me strength at times when I have needed it as a caregiver.
	23. My faith in a higher power (God, angels, Virgin Mary, saints, nature) helps me face the challenges of my daily life as a caregiver.
	24. My connection to God, a higher power, or nature in my caregiving experience has allowed me to strengthen and expand my relationship with the family member I care for.
	25. My participation in religious and/or spiritual practices brings me comfort and support while caring for my loved one.
	26. In times of decision-making, my spirituality is a resource for doing what is best for my family member.
	27. Caring for my family member with chronic disease has led me to reflect on life, death, and my belief system.
	28. During my work as a caregiver, I make time for practices that connect me with a higher power (God, angels, Virgin Mary), such as prayer, meditation, Bible reading, and praying the rosary.
	29. Before beginning my day, I entrust it to a higher power (God, angels, Virgin Mary) so that I can carry out my caregiving activities in the best way possible.
	30. My connection to God, a higher power, or nature during the caregiving experience has allowed me to find positive meaning in caregiving activities.

The assessment process was conducted confidentially, as the judges were not informed of the identities of the other members of the expert panel. An email with an invitation letter and the research project overview was sent to each judge. Upon acceptance, they received the first version of the instrument along with an assessment form, which detailed the conceptual definition of family caregiver transcendence, a description of the instrument development process, and instructions for completing the assessment. 

The experts assessed each item individually regarding clarity, coherence, and relevance. To determine the level of agreement among raters, Kendall's W coefficient was calculated, along with the Content Validity Index (CVI) at both the item level and the overall scale level, as well as Aiken's V coefficient^45^-^48^. 

Items that did not reach an acceptable validity index or for which three or more judges agreed that the item was not relevant or did not align with the construct were eliminated. However, if an item was deemed relevant and consistent with the construct but two or more judges provided similar suggestions to improve clarity, those recommendations were incorporated. Similarly, if four or more judges indicated an item lacked clarity, it was adjusted accordingly^43^. 

All observations related to clarity, coherence, and relevance were compiled into an Excel matrix. Based on consensus among the researchers, recommendations deemed essential by the raters for improving item and the phenomenon comprehension were considered. As a result of this process, the second version of the instrument was developed^43^,^46^. 

The second version of the instrument and the statistical analysis were then emailed to the 15 raters. They were asked whether a second round of evaluation was necessary, whether they had any comments on the new version, and whether the instrument met the criteria of clarity, coherence, and relevance to proceed with pilot testing. Of the 15 raters, 13 responded that that an additional round was not necessary, based on the results obtained. 

**Phase 5. First pilot testing**

The second version of the instrument was administered to 30 family caregivers of individuals with chronic diseases to assess item clarity and conduct a preliminary reliability analysis^43^,^46^. The instrument was applied in the waiting area of the outpatient service at Juárez Hospital in Mexico City. The following eligibility criteria were considered: 

**Inclusion criteria**

- Men and women aged 18 years or older.

- Family caregivers providing either basic care (assistance with activities of daily living such as bathing, dressing, feeding, repositioning, and ambulation) and/or specialized care (administering parenteral medications, monitoring vital signs, administering enteral nutrition, fluid monitoring, or other advanced caregiving activities) to a chronically ill person.

- Family caregivers with a minimum of six months of experience providing care to a chronically ill person with some level of dependency (Barthel Index score ≥ 40).

**Statistical analysis**

Frequencies and percentages were used to describe raters' characteristics, their assessments of clarity, coherence, and relevance, as well as the sociodemographic data of participants in the pilot test. 

The Content Validity Index (CVI) was calculated at both the item level (I-CVI) and the overall scale level. This index evaluates the relevance and representativeness of each item in relation to the theoretical definition of the construct. Calculations were performed in Excel. To determine the I-CVI, the number of experts who rated an item as either 3 or 4 was divided by the total number of experts. The overall content validity was obtained by summing all I-CVIs scores and dividing by the total number of items. A CVI value of ≥ 0.90 was considered indicative of optimal content validity^45^. Additionally, Aiken's V was used to assess content validity further. This calculation was also performed in Excel (version 2409) using the formula: V= x̅ - l / k, where x̅ is the mean of the judges' ratings, l represents the lowest rating given by the judges, and k is the range of possible scores (in this case, from 1 to 4). A critical value of V = 0.70 was established as the threshold, based on the number of raters and rating categories, as well as a p-value > 0.05; confidence intervals were also calculated^47^. 

Kendall's W coefficient of concordance was used to assess the level of agreement among raters, with a significance level of p < 0.05 indicating the existence of statistically significant concordance^48^. This analysis was performed using SPSS statistical software, version 25. 

The instrument's preliminary overall reliability was assessed by administering the second version to a sample of 30 caregivers of individuals with chronic illnesses. For this purpose, two reliability coefficients—Cronbach's alpha and McDonald's omega—were used to obtain robust and comparable estimates of the instrument's internal consistency. 

All collected data, including the assessment form and supplementary materials, are available in Mendeley Data for free access and consultation^49^. 

**Ethical and legal considerations**

This study complied with the official Mexican standard NOM-012-SSA3-20, which establishes criteria for conducting health research involving human subjects, as well as the General Health Law Regulations on Health Research, the Declaration of Helsinki, and the Nursing Code of Ethics. Informed consent was obtained from all pilot test participants after they received an explanation of the research, and the corresponding instruments were provided thereafter. The research protocol was approved by the Ethics and Research Committee of Hospital Juárez de México (Registration No. HJM 006/24-1). 

## Results

**Characteristics of participating judges**

Of the 15 judges, five were men, and ten were women. Nine were from Mexico and six from Colombia, with a mean age of 45 years (range: 36 to 60 years). Eleven held a doctoral degree in Nursing or Nursing Science, while four held a master's degree in Nursing or related educational fields. On average, the judges had 16 years of professional and research experience. 

**Content validity indices**

The clarity of the items was rated by the judges using two categories: "clear, but requires minor modifications" and "clear and does not require modifications," indicating that the items were generally perceived as clear. Regarding coherence, all items—except item nine, which one expert rated as "poorly coherent"—were assessed as "moderately coherent" or "coherent." All 30 items were rated as either "quite relevant" or "very relevant," resulting in a content validity of 1.00 for each item and an overall scale-level content validity index of 1.00 as well. These results indicate that the instrument demonstrates relevance and optimal content validity. 

**Aiken's V**

As shown in Table 2, Aiken's V values range from 0.66 to 1, indicating a moderate to high range of content validity. According to the conventional interpretation of this index, values equal to or greater than 0.70 are generally considered acceptable. However, there are certain items where Aiken's V is lower than this threshold, with items 4, 5, 6, 7, 10, and 27 standing out, especially in the criteria of clarity and coherence, with values ranging between 0.66 and 0.68. These results suggest that these items may require revision or reformulation to improve clarity and consistency. In contrast, other items, such as item 30, have perfect Aiken's V values (1) in all three criteria, indicating excellent acceptance by the judges. The lower and upper confidence limits also support these findings, indicating consistency in the assessments. 

**Table 2:** Aiken's V for raters' assessment

Items	Criterion	Aiken's V	95% CI
1. My experience as a caregiver has allowed me to get to know myself better.
	Clarity	0.71	0.56 - 0.82
	Coherence	0.82	0.68 - 0.90
	Relevance	0.71	0.56 - 0.82
2. Providing care to a chronically ill person has led me to learn more about my emotions and feelings.
	Clarity	0.73	0.59 - 0.84
	Coherence	0.75	0.61 - 0.85
	Relevance	0.75	0.61 - 0.85
3. My experience as a caregiver has helped me find purpose in my life.
	Clarity	0.71	0.56 - 0.82
	Coherence	0.71	0.56 - 0.82
	Relevance	0.75	0.61 - 0.85
4. Caring for a chronically ill person has led me to reflect on my life and take care of my health.
	Clarity	0.66	0.52 - 0.78
	Coherence	0.66	0.52 - 0.78
	Relevance	0.71	0.56 - 0.82
5. In my experience as a caregiver, I have learned to value my life more.
	Clarity	0.68	0.54 - 0.80
	Coherence	0.68	0.54 - 0.80
	Relevance	0.73	0.59 - 0.84
6. Caring for a chronically ill person has led me to learn more about my own needs.
	Clarity	0.66	0.52 - 0.78
	Coherence	0.66	0.52 - 0.78
	Relevance	0.71	0.56 - 0.82
7. I have learned to manage stressful, distressing, or emotionally charged situations associated with caregiving.
	Clarity	0.66	0.52 - 0.78
	Coherence	0.68	0.54 - 0.80
	Relevance	0.71	0.56 - 0.82
8. I allow myself to take breaks or time off without feeling guilty
	Clarity	0.71	0.56 - 0.82
	Coherence	0.73	0.59 - 0.84
	Relevance	0.71	0.56 - 0.82
9. I regularly make time for activities that benefit me personally.
	Clarity	0.71	0.56 - 0.82
	Coherence	0.73	0.59 - 0.84
	Relevance	0.73	0.59 - 0.84
10. I have learned to deal constructively with the challenges I may face as a caregiver.
	Clarity	0.68	0.54 - 0.80
	Coherence	0.71	0.56 - 0.82
	Relevance	0.71	0.56 - 0.82
11. Thanks to the caregiving experience, I have been able to improve my relationship with my family member with chronic disease.
	Clarity	0.71	0.56 - 0.82
	Coherence	0.71	0.56 - 0.82
	Relevance	0.73	0.59 - 0.84
12. I enjoy sharing with my family member with chronic disease.
	Clarity	0.71	0.56 - 0.82
	Coherence	0.71	0.56 - 0.82
	Relevance	0.73	0.59 - 0.84
13. Communication with my family member is clear and good all the time.
	Clarity	0.71	0.56 - 0.82
	Coherence	0.71	0.56 - 0.82
	Relevance	0.73	0.59 - 0.84
14. I treat my family member with respect at all times.
	Clarity	0.71	0.56 - 0.82
	Coherence	0.71	0.56 - 0.82
	Relevance	0.73	0.59 - 0.84
15. Despite the illness, I make plans for the future with my family member.
	Clarity	0.73	0.59 - 0.84
	Coherence	0.73	0.59 - 0.84
	Relevance	0.73	0.59 - 0.84
16. I try to take care of my family member to the best of my ability.
	Clarity	0.71	0.56 - 0.82
	Coherence	0.71	0.56 - 0.82
	Relevance	0.73	0.59 - 0.84
17. The emotional and spiritual support I provide to my family member is constant.
	Clarity	0.73	0.59 - 0.84
	Coherence	0.73	0.59 - 0.84
	Relevance	0.75	0.61 - 0.85
18. I understand the needs that my family member with chronic disease may have.
	Clarity	0.71	0.56 - 0.82
	Coherence	0.71	0.56 - 0.82
	Relevance	0.73	0.59 - 0.84
19. Despite the difficult and vulnerable moments I may experience as a caregiver, I strive to maintain a personal, stable, and harmonious relationship with my family member.
	Clarity	0.71	0.56 - 0.82
	Coherence	0.71	0.56 - 0.82
	Relevance	0.73	0.59 - 0.84
20. I feel emotionally close and connected to my family member with chronic disease, which motivates me to provide better care.
	Clarity	0.71	0.56 - 0.82
	Coherence	0.71	0.56 - 0.82
	Relevance	0.73	0.59 - 0.84
21. Caring for another person has strengthened my connection to God, a higher power, or nature.
	Clarity	0.75	0.61 - 0.85
	Coherence	0.71	0.56 - 0.82
	Relevance	0.77	0.63 - 0.87
22. My spirituality has given me strength at times when I have needed it as a caregiver.
	Clarity	0.73	0.59 - 0.84
	Coherence	0.73	0.59 - 0.84
	Relevance	0.73	0.59 - 0.84
23. My faith in a higher power (God, angels, Virgin Mary, saints, nature) helps me face the challenges of my daily life as a caregiver.
	Clarity	0.73	0.59 - 0.84
	Coherence	0.73	0.59 - 0.84
	Relevance	0.73	0.59 - 0.84
24. My connection to God, a higher power, or nature in my caregiving experience has allowed me to strengthen and expand my relationship with the family member I care for.
	Clarity	0.71	0.56 - 0.82
	Coherence	0.71	0.56 - 0.82
	Relevance	0.71	0.56 - 0.82
25. My participation in religious and/or spiritual practices brings me comfort and support while caring for my loved one.
	Clarity	0.71	0.56 - 0.82
	Coherence	0.71	0.56 - 0.82
	Relevance	0.71	0.56 - 0.82
26. In times of decision-making, my spirituality is a resource for doing what is best for my family member.
	Clarity	0.71	0.56 - 0.82
	Coherence	0.71	0.56 - 0.82
	Relevance	0.71	0.56 - 0.82
27. Caring for my family member with chronic disease has led me to reflect on life, death, and my belief system.
	Clarity	0.69	0.54 - 0.80
	Coherence	0.69	0.54 - 0.80
	Relevance	0.69	0.54 - 0.80
28. During my work as a caregiver, I make time for practices that connect me with a higher power (God, angels, Virgin Mary), such as prayer, meditation, Bible reading, and praying the rosary.
	Clarity	0.69	0.54 - 0.80
	Coherence	0.69	0.54 - 0.80
	Relevance	0.69	0.54 - 0.80
29. Before beginning my day, I entrust it to a higher power (God, angels, Virgin Mary) so that I can carry out my caregiving activities in the best way possible.
	Clarity	0.69	0.54 - 0.80
	Coherence	0.69	0.54 - 0.80
	Relevance	0.69	0.54 - 0.80
30. My connection to God, a higher power, or nature during the caregiving experience has allowed me to find positive meaning in caregiving activities.
	Clarity	1	0.92 - 1
	Coherence	1	0.92 -1
	Relevance	1	0.92 -1

**Concordance of judges' assessments**

Kendall's W coefficient of concordance for the dimensions of clarity, coherence, and relevance yielded a p<0.001, indicating a highly significant level of agreement among the expert judges assessments. 

The assessment form provided to the judges included a specific section to assess the instrument's adequacy. In this section, judges were asked whether they considered the number of items per dimension sufficient. If not, they were invited to indicate which dimension required additional items and to suggest other indicators or items that could contribute to the instrument. As a result, 100% of the raters confirmed that the number of dimensions and items was sufficient, supporting the robustness of our proposal. 

**Second version of the instrument**

According to the experts' assessments, the most recurrent recommendations focused on minor adjustments in some words or the wording of certain items. Following review and consensus among the researchers, these modifications were implemented without altering the core meaning of the items, fulfilling the revision requirements associated with Aiken's V statistic while maintaining the relevance ratings assigned by the judges (quite relevant and very relevant). The revised items, along with the second version of the Transcend instrument, are included as supplementary material and are freely available for consultation in the Mendeley Data repository^49^. 

**Pilot testing**

A pilot test was conducted with 30 caregivers of individuals with chronic diseases. Of the participants, 73.33% were women, with a mean age of 47 years (range: 26–75 years). In addition to their caregiving responsibilities, 60% were employed part-time. On average, participants had been caring for their family member for 8.7 years (range: 1–20 years), dedicating approximately 116 hours per week to caregiving tasks. 

In terms of kinship to the care recipient, 33.33% of participants cared for their mother, 20.00% for their daughter, 20.00% for their spouse (husband or wife), and 26.67% for another type of relative. Regarding the primary diagnosis of the person receiving care, 56.66% had diabetes, 26.67% had arterial hypertension, 10.00% had chronic kidney disease, and 6.67% were caring for someone with another chronic condition. 

The second version of the instrument was assessed for preliminary reliability, yielding a Cronbach's alpha coefficient of 0.90 and a McDonald's omega coefficient of 0.88. 

## Discussion

In this research, family caregiver transcendence was defined as a reflective and spiritual human capacity that enables caregivers to transform aspects of the caregiving experience at the intrapersonal, interpersonal, and transpersonal levels, thereby facilitating the attainment of a state of psycho-spiritual well-being. 

According to the Royal Spanish Academy (Real Academia Española, RAE), the term 'transcendence' derives from the Latin transcendentia and refers to "that which goes beyond natural limits."^50^ Webster's New Universal Unabridged Dictionary defines "transcend" as the ability to be transcendent, surpassing ordinary boundaries, extending beyond the limits of human experience, and existing beyond comprehension^51^. 

The definitions of transcendence presented in this research and the consulted dictionaries share a common essence: both emphasize the capacity to go beyond the ordinary. Family caregiver transcendence is understood as a profound transformation occurring at the intrapersonal, interpersonal, and transpersonal levels, leading to a state of psycho-spiritual well-being. In contrast, the definitions offered by the RAE and Webster's Dictionary emphasize the idea of surpassing natural limits and the boundaries of comprehension. The key distinction lies in the fact that caregivers' definition focuses on a practical and experiential process related to the act of caring, while the dictionary definitions emphasize a more abstract quality of transcendence. Despite these differences, both perspectives converge in their recognition of transcendence as an expansion beyond conventional limits. 

This phenomenon is associated with end-of-life events and arises from a prior state of sensitization or heightened awareness of human vulnerability. Amid one's own or others' vulnerability, transcendence occurs, and it is this very thing that makes transcendence the reflection of a human capacity to extend the self beyond the common limits of the immediate context, opening the way to new perspectives and experiences^23^. 

In the caregiving experience, family caregivers —due to their commitment to their ill relative—may lose their personal connection with themselves to the point of no longer recognizing their identity or neglecting their own self-care. This disconnection can lead to periods of vulnerability. However, as caregivers gain experience, the caregiving organization improves and expands in response to these initial challenges. Finally, a transformation occurs in the caregiver's life as a result of this experience (transcendence), which enables a shift in how they think, act, and respond to the ongoing challenges and circumstances of caregiving^52^. 

The concept of family caregiver transcendence is grounded in the post-empiricist philosophical tradition, as it allows us to see human beings from their context and history^53^. This perspective provides nursing with a comprehensive view of transcendence, framing it as the outcome of the caregiving experience, influenced by the individual, familial, social, and spiritual context in which it unfolds. This view is based on the transformative unitary paradigm, which upholds the idea that human beings and their environment form an inseparable unity^54^. 

In this study, the expert judges determined that all 30 items had a high level of relevance, resulting in an overall CVI of 1.00, indicating an excellent level of content validity^45^. These findings are consistent with those reported by Kor et al.^55^, who designed and validated a health literacy scale for family caregivers of chronically ill older adults. In their study, relevance was assessed by seven expert judges, and both the overall and individual CVI scores for each item were ≥ 0.90. 

These results are consistent with the recommendations in the literature, which suggests that a content validity index (I-CVI) between 0.78 and 1 is satisfactory for ensuring the instrument' validity. Using a systematic validation process and achieving satisfactory content validity indices reinforce the quality of the instrument developed in this study. According to Yusoff^56^, this approach ensures that the inferences drawn from the instrument are valid and applicable, providing a solid foundation for its use in the context of family caregivers —similar to findings reported in studies involving populations with similar characteristics. 

According to Aiken^57^, the interpretation of the V coefficient is based on both its magnitude and the determination of statistical significance using critical value tables. Aiken's V values equal to or greater than 0.69 are considered acceptable to affirm adequate content validity when there are 15 raters and four rating categories. However, it is important to interpret these values in conjunction with the upper and lower confidence interval limits. 

In this study, the overall Aiken's V value was 0.72 for the criteria of clarity and coherence and 0.73 for relevance. However, at the individual level, some items —specifically items 4, 5, 6, 7, 10, and 27—did not reach the acceptable threshold of 0.69, with values ranging from 0.66 to 0.689, particularly in the clarity and coherence dimensions. As a result, the judges’ recommendations were taken into account to improve the clarity and consistency of these items. 

The 95% confidence intervals provide information about the stability of Aiken's V estimates within the population. According to Merino-Soto and Livia-Segovia^58^, these confidence intervals represent the range within which the true value of V is highly likely to fall—typically with 95% certainty or based on a different critical value selected by the researcher. 

The use of confidence intervals not only allows for verifying the statistical significance of Aiken’s V coefficient but also helps assess whether the magnitude of the coefficient is sufficiently high to meet validity standards. This is particularly useful when determining whether an item should be retained or revised. In the initial stages of instrument development, a more liberal threshold—such as an Aiken's V of 0.50—may be acceptable^59^. However, as the process advances, particularly if a second round of expert review is conducted, it is advisable to apply stricter criteria, such as a minimum Aiken's V of 0.70, to ensure that retained items meet the more stringent levels of content validity. 

For this reason, all items were reviewed, and modifications were made based on the judges' most recurrent comments. While most of the raters indicated that the 30 items were clear, they recommended minor modifications. In response to these suggestions, items 1 through 29 were revised with minor adjustments, ensuring that each item's main idea remained intact to preserve its relevance. 

In contrast, item 30 achieved a perfect Aiken's V value across all three assessment criteria, indicating excellent acceptance by the judges and suggesting that no modifications were required. These results are consistent with the confidence intervals, which confirm the stability and consistency of the experts' assessments. This consistency further reinforces the reliability of the results obtained^57^-^59^.

Kendall's W coefficient of concordance measures the level of association among judges' ratings by assessing the level of agreement of their opinions. This coefficient only reflects the agreement among raters without indicating the direction of their assessments. Therefore, judges may agree on whether or not the items are clear, coherent, or relevant. The significance value associated with Kendall's W serves as an indicator of the agreement's robustness, providing confidence in the validation process. In many cases, a p-value less than 0.05 is often considered sufficient to establish statistically significant concordance and validity^48^,^60^. 

Kendall's W coefficient of concordance analysis showed a p < 0.001 for clarity, consistency, and relevance in the present study. The value of p <0.001 is considerably lower than the standard threshold of p < 0.05, which reinforces the reliability of the observed agreement and provides greater confidence in the instrument's content validity. Furthermore, the Cronbach's alpha values obtained for each dimension reinforce these findings, showing high levels of internal consistency in the assessments, suggesting that the assessments were stable and coherent among the judges. This level of internal consistency is crucial to ensure that the instrument accurately measures the constructs of interest, as it minimizes unwanted variability in the assessments. 

The validity indices should be interpreted alongside the concordance indices, as both allow for determining the level of content validity based on expert judgment. When validity indices are acceptable, and concordance indices indicate significant agreement, it can be concluded that the instrument’s content adequately and representatively reflects the construct it aims to measure. In this case, the construct evaluated is family caregiver transcendence, assessed through the intrapersonal relationship, the connection with the care recipient, and the transpersonal relationship, which includes the caregiver’s connection with nature. 

Both similarities and discrepancies can be identified in instruments that assess transcendence or related constructs. Most of these instruments explore transcendence based on a theoretical or conceptual framework, adding interpretative richness to their use (something similar to the present instrument). However, they often focus mainly on the transpersonal connection, the divine, or the mystical, leaving aside other dimensions of the human being^37^,^38^. In contrast, the instrument developed evaluates transcendence encompassing all three dimensions of being: intrapersonal, interpersonal, and transpersonal. 

Pamela Reed^61^-^63^ points out that the broadening or strengthening of these dimensions leads human beings toward transcendence, which in turn promotes states of mental well-being. These states manifest through feelings of fullness, meaning, fulfillment, and mental health. 

**Contribution to nursing discipline**

This research provides an initial conceptual and methodological approach to the study of transcendence in the context of family caregiving, based on Pamela Reed's theory of self-transcendence, a theoretical framework widely used in scientific nursing literature and applicable across diverse populations and moments and situations of life^64^. By doing so, it contributes to the theoretical and scientific knowledge of the nursing discipline. Additionally, this study proposes a tool designed to assess, from the caregiver’s experience, the relationship with oneself, with the care recipient, and the link to a transcendental dimension or higher power. 

The “Transcend” measurement instrument may serve as a valuable tool in future research aimed at understanding the importance of transcendence in the mental health and well-being of caregivers of individuals with chronic illness. This tool would also make it possible to assess the intrapersonal, interpersonal, and transpersonal dimensions of caregivers, thereby facilitating the design of comprehensive, holistic care plans tailored to their needs. 

## Conclusions

The concept of family caregiver transcendence in the context of chronic illness is grounded in Pamela Reed's theory of self-transcendence. This idea is framed within the post-empiricist philosophical tradition and the transformative unitary paradigm, allowing for a comprehensive approach to the phenomenon of caregiving in chronic disease contexts. From this perspective, the essential elements of the caregiver's being are explored, considering their context and personal history, thereby facilitating the integration of mental and spiritual aspects into their caregiving experience. 

The "Transcend" instrument has demonstrated adequate content validity and high preliminary reliability, suggesting its potential as a valuable tool for assessing transcendence in family caregivers of individuals with chronic illness. However, a second phase of the project is still needed to determine its construct validity and reliability —overall and by dimensions— to consolidate it as an assessments instrument. 
